# YOLOv5-MS: Real-Time Multi-Surveillance Pedestrian Target Detection Model for Smart Cities

**DOI:** 10.3390/biomimetics8060480

**Published:** 2023-10-09

**Authors:** Fangzheng Song, Peng Li

**Affiliations:** 1 College of Information Science and Engineering, Henan University of Technology, Zhengzhou 450001, China; 2021930814@stu.haut.edu.cn; 2 Beijing Institute of Technology, Beijing 100081, China

**Keywords:** smart city, multi-surveillance, SE model, YOLOv5-MS, IoU loss, image augmentation

## Abstract

Intelligent video surveillance plays a pivotal role in enhancing the infrastructure of smart urban environments. The seamless integration of multi-angled cameras, functioning as perceptive sensors, significantly enhances pedestrian detection and augments security measures in smart cities. Nevertheless, current pedestrian-focused target detection encounters challenges such as slow detection speeds and increased costs. To address these challenges, we introduce the YOLOv5-MS model, an YOLOv5-based solution for target detection. Initially, we optimize the multi-threaded acquisition of video streams within YOLOv5 to ensure image stability and real-time performance. Subsequently, leveraging reparameterization, we replace the original BackBone convolution with RepvggBlock, streamlining the model by reducing convolutional layer channels, thereby enhancing the inference speed. Additionally, the incorporation of a bioinspired “squeeze and excitation” module in the convolutional neural network significantly enhances the detection accuracy. This module improves target focusing and diminishes the influence of irrelevant elements. Furthermore, the integration of the K-means algorithm and bioinspired Retinex image augmentation during training effectively enhances the model’s detection efficacy. Finally, loss computation adopts the Focal-EIOU approach. The empirical findings from our internally developed smart city dataset unveil YOLOv5-MS’s impressive 96.5% mAP value, indicating a significant 2.0% advancement over YOLOv5s. Moreover, the average inference speed demonstrates a notable 21.3% increase. These data decisively substantiate the model’s superiority, showcasing its capacity to effectively perform pedestrian detection within an Intranet of over 50 video surveillance cameras, in harmony with our stringent requisites.

## 1. Introduction

Fueled by technological advancements and a growing emphasis on security awareness, video surveillance has garnered extensive adoption across smart cities [1,2]. Nevertheless, the abundance of surveillance images demands increased labor costs, a process that consumes time and fails to ensure the precision of pedestrian identification. The integration of intelligent target detection models rooted in deep learning into smart city contexts holds promise for enhancing urban security systems [3], thereby reducing the property damage and casualties caused by criminals with a lower cost and higher efficiency. This model can be applied to 24 h pedestrian target detection in smart cities thanks to the creation of a comprehensive dataset that enhances its adaptability to various scenarios. This model also exhibits distinctive characteristics in nighttime recognition. By implementing this approach, it becomes possible to alert security personnel only when a relevant target is detected, thereby greatly improving their efficiency and ensuring the area’s security.

In smart city environments abundant with cameras, video capture devices are frequently connected to the city’s intelligent system through 4G or 5G networks. This integration enables the smart city’s security monitoring management module to remotely access real-time video feeds, facilitating the vigilant monitoring of day-to-day security threats within the urban environment. Nonetheless, numerous network factors necessitate thorough consideration. As an illustration, within the context of our smart park experiment, we deployed a total of 220 cameras. However, owing to the constraints related to hardware performance and network bandwidth, conducting simultaneous experiments with all 220 cameras was not feasible. Consequently, we selected 50 cameras for real-time video stream retrieval, which were then uploaded to the cloud for experimentation. Notably, our findings indicate that this operation demanded a network bandwidth of up to 183 Mbps. Extrapolating from this result, it became evident that to accommodate the retrieval of video streams from all 220 cameras, a network bandwidth of approximately 805 Mbps would be required. The substantial uplink speeds entail significant expenses, and a substantial portion of the video streams end up as redundant resources. Consequently, we witnessed the emergence of edge computing-driven target detection algorithms and hardware solutions [4]. In recent times, Nvidia has unveiled a suite of edge computing-based target detection computing platforms exemplified by Jetson Xavier, Jetson Nano, and Jetson Xavier AGX [5,6]. However, a notable discrepancy arises between the cost and performance of these computing platforms. Moreover, their capabilities are typically confined to managing a limited number of camera video streams. This limitation proves incongruent with the demands of target detection across the expansive networks of large-scale cameras. The exigency of addressing these challenges has spurred the necessity for a novel solution. Within this paper, we introduce a groundbreaking scheme involving the incorporation of high-performance CPUs and GPUs into the Intranet of the smart city infrastructure. Here, the CPU is tasked with the efficient retrieval of multiple camera video streams through multi-threading. In parallel, the GPU undertakes the computation of the target detection model and subsequently transmits the computed results to the management system. This methodology, which involves processing within the Intranet and subsequently uploading the results to the extranet, offers a substantial reduction in network costs while enabling concurrent detection across multiple video streams. Lastly, we undertake the optimization of the Yolov5s model, customizing it to be better aligned with the demands of pedestrian target detection in smart cities. This endeavor culminates in a well-balanced alignment of accuracy, speed, and cost considerations.

In the domain of deep learning, two fundamental types of target detection models exist. The first type entails a network model that performs region proposals prior to carrying out target detection. This approach is distinguished by networks with an exceptional detection accuracy, albeit at the expense of a slower computing speed, for example, Mask R-CNN [7], Fast R-CNN [8], and Faster R-CNN [9,10]. The second type is a single-stage detection model, which operates with a single neural network from input to output. This approach ensures swift inference without the need for generating candidate frames. For example, YOLO [11,12,13,14,15] and SSD [16,17]. This paper employs the YOLO-based target detection algorithm to achieve real-time target detection across multiple cameras. In the context of a smart city, objects situated farther from the camera encompass smaller pixels. Additionally, smart cities host a wealth of information and are prone to greater occlusions. Consequently, target detection within the smart city domain represents a significant and valuable research endeavor.

When delving into target detection within urban environments, some scholars have undertaken improvements to existing detection models. Bodla et al. [18] elevated the count of prediction frames by adapting the target prediction frame fraction strategy, thereby enhancing the performance concerning occluded targets. However, the potential for better generalization remains a point of consideration. XUE et al. [19] introduced an innovative real-time pedestrian detection algorithm, the multimodal attention fusion YOLO. This algorithm adeptly adjusts to pedestrian detection efficacy during nighttime using the Darknet53 framework. It establishes a loss function, alongside generating anchor frames through the application of the K-means algorithm. The outcomes of their study underscore the method’s effectiveness, as it achieves a notable enhancement in pedestrian detection. PUSTOKHINAIV et al. [20] put forth a strategy that merges Faster-RCNN with a hybrid Gaussian model within intricate backgrounds. This approach aims to eliminate the impact of video backgrounds on images, elevate the image resolution, and ultimately, enhance the overall effectiveness of detecting pedestrian targets. Nonetheless, this approach encounters challenges linked to substantial model sizes and extended training periods. HSU et al. [21] pioneered a ratio-aware mechanism to fine-tune the aspect ratio of images, effectively addressing false target pedestrian detections through the segmentation of the initial image. This method notably enhances the accuracy of target pedestrian detection. However, challenges persist, particularly in rectifying instances of occluded and overlapping pedestrians that result in both missed detections and erroneous identifications.

In summary, substantial progress has been achieved in pedestrian target detection within urban environments. However, the majority of research efforts have been directed towards enhancing model performance through scaling up the model’s size [22]. This approach, while seeking improvement, introduces challenges such as a sluggish detection speed, extensive model scale, and elevated hardware costs, leading to diminished practicality. Within this paper, we address and rectify these challenges, delving into the YOLOv5 model, while concurrently aligning with our specific requirements. Our investigation culminates in the creation of the YOLOv5-MS target detection model. By optimizing the model’s BackBone structure, we manage to reduce its size, thereby bolstering the inference speed. The integration of the SE module within the network enhances the detection accuracy. Furthermore, we employ the K-means technique to generate varied-sized prior frames and amplify the performance using the Retinex image enhancement algorithm. Ultimately, the incorporation of the Focal-EIOU loss calculation method strengthens the alignment between the context of this experimental study and the YOLOv5 algorithm, leading to a more seamless integration.

## 2. Models

### 2.1. Optimized Video Stream Acquisition Method

**Figure 1:**
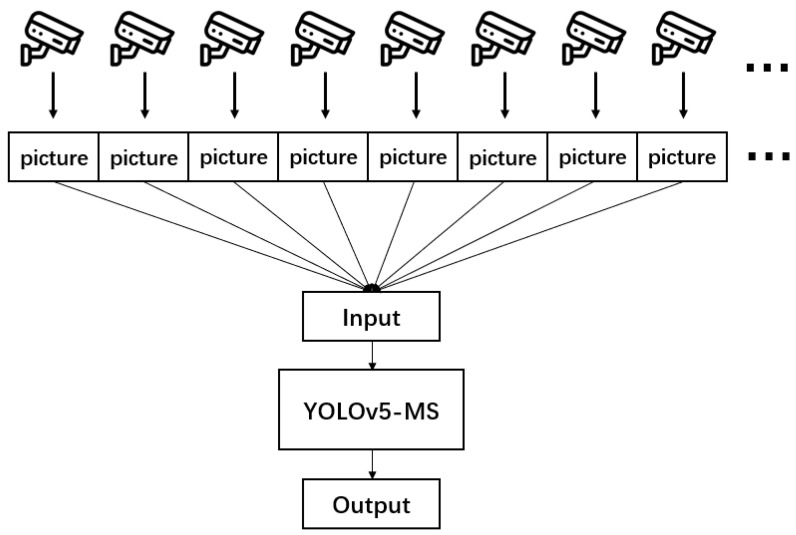
Multi-camera video push flow to YOLOv5-MS.

In the YOLOv5 model, images are acquired by simultaneously fetching multi-camera video streams through multiple threads. Subsequently, the model proceeds to perform inference by separately detecting each frame captured with the cameras. This approach gives rise to several challenges: Firstly, when hardware capabilities fall short of fulfilling real-time detection requirements, image detection may experience delays. As the backlog of images accumulates, it could lead to target detection referring to images from hours ago, consequently failing to uphold real-time detection standards. Secondly, excessive image accumulation can strain memory resources, undermining the model’s stability. Lastly, if a specific video stream experiences a prolonged delay, it might lead to automatic disconnection from the model, thereby affecting the model’s detection process for that particular camera. In light of the scenarios outlined above, when detecting numerous camera images, this paper addresses these challenges through enhancements in camera video image acquisition. By employing a multi-threaded strategy, each individual thread undertakes the acquisition of a camera’s video stream. The role of each thread involves a continuous extraction of the latest frames from its respective camera. This separation ensures that during model image detection, a steady stream of the latest images is available. Module separation enables the model to detect the most recent real-time images after completing the previous round of image detection. This capability fulfills the real-time target detection requirements of large-scale cameras. A visual representation of the video stream reception method is depicted in Figure 1.

By employing this approach, the aforementioned challenges can be effectively mitigated, enabling the achievement of real-time processing for camera video streams even in scenarios involving extensive detection. The detection performance of this method relies on the capacity of the computer hardware. If it can detect every frame from 50 cameras within 0.5 s, this means two real-time images per camera can be detected in one second. The distinction between detecting thirty frames or only two frames in one second has a negligible effect on the results. As a result, in this experiment, the proposed method is utilized to capture a wide array of camera streams, allowing the model to simultaneously process up to 50 or even 100 camera streams at the highest level of efficiency.

### 2.2. YOLO Basic Principle

The primary aim of this research is to achieve pedestrian target detection within smart cities to enhance urban security. With the deployment of the model at the edge, real-time responsiveness is of paramount importance. Furthermore, considering potential extended operational durations, both the model’s mean average precision and stability emerge as vital considerations. The Yolo series effectively satisfies these prerequisites. The YOLO model idea is to treat the detection of objects as a regression problem and predict the input images as detection frames and target class probabilities using a neural network. The YOLOv1 model is the initial version of the whole YOLO series. The idea is first to transform the image into having a 448 × 448 resolution and divide it into 7 × 7 cells, predicting the confidence and category scores of the boxes based on the position and content of each cell.

**Figure 2:**
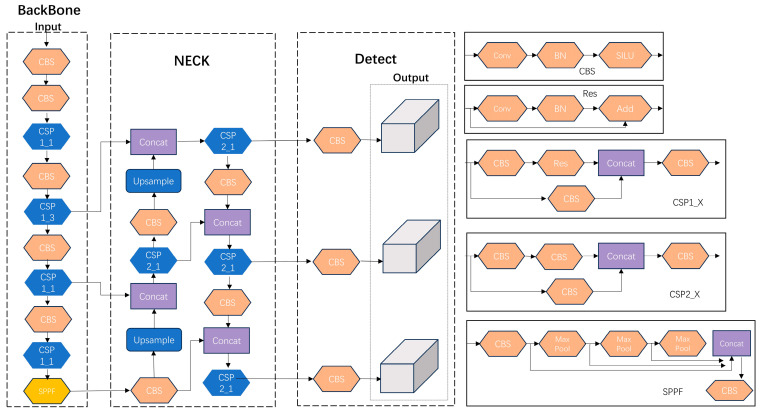
YOLOv5 network structure.

YOLOv5 is a contemporary and GPU-optimized target detection model. Within the current YOLOv5 framework, distinct variants exist, namely YOLOv5s, YOLOv5n, YOLOv5l [23], YOLOv5m, and YOLOv5x [24]. Among this array of network models varying in size, YOLOv5s stands out due to its purposeful network simplification, which is tailored for edge deployment to facilitate real-time target detection. Furthermore, YOLOv5s boasts notable advantages, including a heightened detection stability, accuracy, and simplified deployment. Its performance surpasses those of YOLOv5n, YOLOv7, and YOLOv8 in specific scenarios, rendering it an optimal selection for undertaking comprehensive inspections using large-scale camera systems. YOLOv5s comprises three primary components: the Neck, the Backbone, and the prediction. The effective interplay between these distinct modules contributes to its commendable performance. YOLOv5 introduces several enhancements, encompassing Mosaic data augmentation at the input stage along with adaptive anchor frame computation [25]. The Backbone component incorporates the focus and CSP_X structures, while the Neck segment integrates the CSP2 structure, all working cohesively to amplify the amalgamation of network features [26]; the use of spatial pyramidal pooling to fuse different sensory fields; the CIOU loss is utilized as the loss function of the bounding box [27]; and the overlapping targets are improved via NMS non-maximal suppression [28]. The structure of YOLOv5 is shown in Figure 2.

### 2.3. Backbone Structure

In complex settings, the diverse shooting angles of cameras capture pedestrians of varying sizes. Directly utilizing the YOLOv5’s algorithm Backbone, network extraction results in slowness and an increased likelihood of missing or misidentifying instances. Consequently, to enhance the recognition and detection of pedestrians spanning various target sizes, refinement is applied to the convolutional layer of the Backbone. Leveraging the reparameterization concept from RepVGG [29], the original convolution within the Backbone is substituted with RepvggBlock. This replacement effectively curtails the channel count within the convolutional layer. RepVGG builds upon the conventional VGG architecture, while incorporating a residual structure [30]. It introduces varied training approaches based on distinct principles governing model training and inference phases. The fundamental notion driving RepVGG involves augmenting model performance by integrating a multi-branch structure into the training network through structural reparameterization.

**Figure 3:**
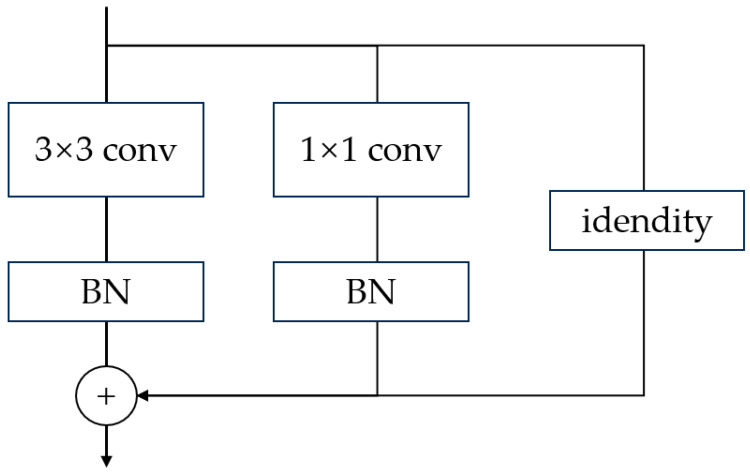
RepvggBlock structure.

The RepvggBlock encompasses Conv3 × 3 + BN (Batch Normalization), Conv1 × 1 + BN, and identity branches. These branches are assigned weights prior to their activation function application in subsequent network iterations. The RepvggBlock configuration is illustrated in Figure 3.

The core idea of RepvggBlock is as follows:

(1) The convolutional layer and BN layer are fused. The parametric calculation of the convolutional layer is shown in Formula (1), where W and B are the weights and biases, respectively. The parameter operation of the BN layer is shown in Formula (2).

(1) Y=W∗X+B

(2) $$Z=Y*\frac{\hat{X}-U}{\sqrt{\sigma^{2}+\vartheta }}+\beta$$

where U and ϑ are the mean and variance, respectively, Y is a learnable parameter, and β is the bias. By substituting the parameters in Formula (1) into Formula (2), the convolutional and BN layers can be combined into one convolutional layer with the bias, as shown in Formula (3).

(3) $$Z=\left(\frac{W}{\sqrt{\sigma^{2}+\vartheta }}*\gamma \right)*X+\frac{B-U}{\sigma^{2}+\vartheta }*\gamma +\beta \;\;$$

**Figure 4:**
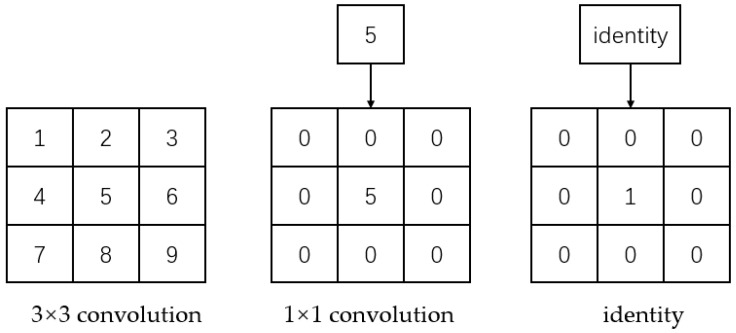
Multi-branch merge process.

(2) The convolutional kernel is transformed into a 3 × 3 scale size. Following the initial step, the model branches into two: a 1 × 1 convolutional layer and an identity branch. For 1 × 1 convolution, the convolution kernel evolves from a 1 × 1 configuration to a 3 × 3 convolutional kernel, with the weight of the null convolution set to 0. In the identity branch, a 3 × 3 convolution kernel is employed with the center weight set to 1 and the remaining weights set to 0. This substitution is depicted in Figure 4. Ultimately, the convolution structure of every branch in the model is transformed into a 3 × 3 convolution. The corresponding weights and biases are subsequently aggregated, amalgamating the branches into a unified 3 × 3 convolution.

Following the principles of network structure reparameterization, the RepvggBlock is skillfully crafted to leverage multiple branches, enhancing the model’s detection efficacy during the training phase. Subsequently, these branches are consolidated into a unified structure for inference, thereby boosting the model’s inference speed. This characteristic aligns seamlessly with the detection requisites of multiple cameras within a smart city context.

### 2.4. Squeeze-and-Excitation Model

In this study, upon the integration of the RepvggBlock module within the Backbone, a notable observation emerges: although the detection speed experiences enhancement, there is a simultaneous decline in detection accuracy. This outcome does not align favorably with the developmental goals of enhancing security within smart city environments. To further enhance the model’s accuracy while satisfying its speed requisites, this study introduces an SE module based on the Backbone structure, aiming to optimize the overall performance [31]. The SE module, which is short for Squeeze-and-Excitation, is a crucial component in deep learning networks. It boosts the power of Convolutional Neural Networks (CNNs) by dynamically adjusting the feature maps during training. By learning how to scale each channel, it helps the network focus on valuable information, improving the performance in tasks like image classification and object detection. Through the incorporation of the SE module to refine functionality, the model achieves synergistic amalgamation. This amalgamated model retains its capability to augment detection performance while upholding a compact model size. This attribute aligns well with the demands of large-scale camera detection within the context of this study.

**Figure 5:**
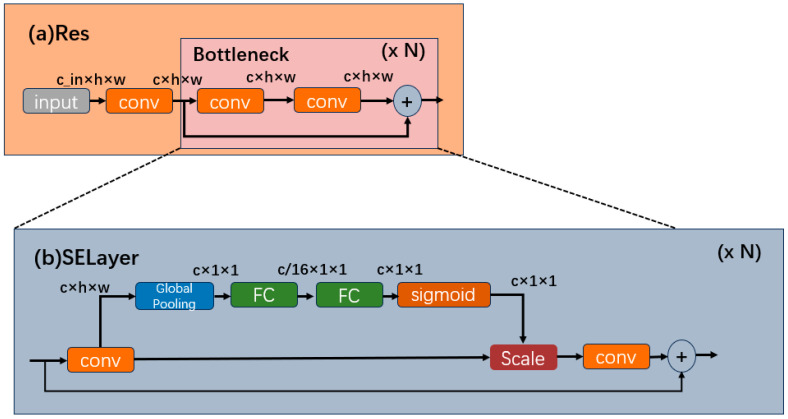
(**a**) Res’s structure of YOLOv5s. (**b**) SELayer structure embed with SE module.

Figure 5 illustrates the incorporation of the SE module, specifically the channel attention module. The SE module aims to obtain more critical feature information utilizing a weight matrix that gives different weights to different image positions from the perspective of the channel domain. It is added to the original residual block via extra pathways. This module employs a global pooling layer to calculate channel weights for the initial channels. Subsequently, these calculated weights undergo refinement for each channel, involving a sigmoid activation function and two fully connected layers. In the final step, the original channels are multiplied by their respective channel weights. Subsequently, during network training, the model’s detection performance is enhanced through gradient descent. The squeezing operation is elucidated in Formula (4).

(4) $$g_{c}=Z_{s}\left(u_{c}\right)\sum_{i=1}^{H}\sum_{j=1}^{W}u_{c}\left(i,j\right),g\epsilon R^{c}$$

where $Z_{s}$ is the squeeze movements and u indicate the input. U∈R H × W × C. c indicates the channel. The excitation operation is shown in Formula (5).

(5) $$t=Z_{e}\left(g,W\right)=\delta (Q_{2}Sigmoid(Q_{1}g))$$

where $Z_{e}$ presents the excitation operation; $Q_{1}$, $Q_{2}\in R^{C\times \frac{C}{r}}$, r indicates the hyperparameter, which is 16, indicating the dimension reduction coefficient of the first fully connected layer. The scale operation is shown in Formula (6).

(6) $$q=Z_{sc}\left(u_{c},t_{c}\right)=u_{c}\times t_{c}$$

where $Z_{sc}$ presents the scale operation.

### 2.5. YOLOv5-MS Structure

**Figure 6:**
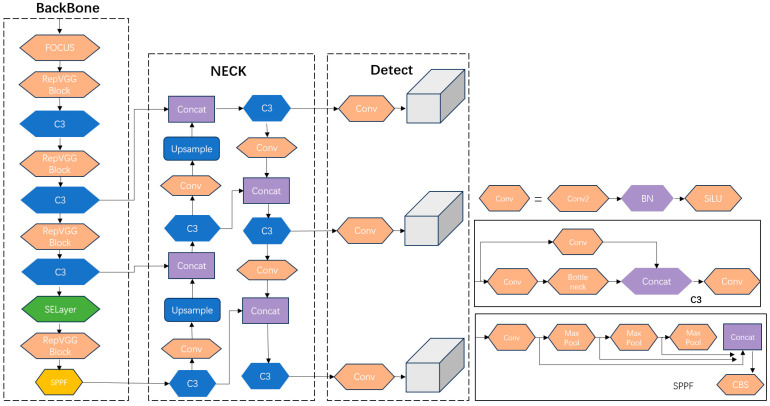
YOLOv5-MS network structure.

The network model structure of YOLOv5-MS is presented in Figure 6. To enhance the model’s detection mAP and speed, YOLOv5s is subjected to the following improvements: (1) incorporating an SE module into the model to enhance its feature extraction capabilities; (2) substituting the original Conv convolution in the model with RepvggBlock to decrease the model’s complexity and increase its detection speed; (3) introducing a focus structure to preserve essential information by reducing the dimensionality, thereby mitigating the risk of model overfitting to some extent.

## 3. Materials and Methods

### 3.1. Dataset

**Figure 7:**
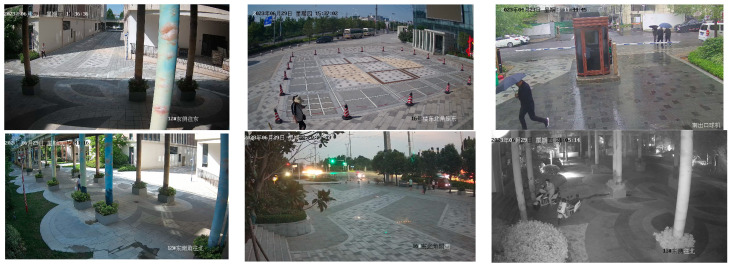
Sample dataset images. The first row shows three different types of weather in the city, sunny, cloudy, and rainy. The second row shows three different times of day: daytime, evening, and nighttime.

Twenty cameras are strategically chosen within the urban landscape, capturing dynamic pedestrian movements to construct the pedestrian dataset for a smart city. The image acquisition process involves capturing a single frame from a live video stream at intervals every 10 s. Image collection was orchestrated across various weather conditions, encompassing sunny, cloudy, nighttime, and rainy periods. This diverse approach was employed to bolster the model’s robustness. The dataset configuration is depicted in Figure 7. The image resolutions vary contingent on the camera specifications, with the camera models encompassing gun and dome variants. Given the prevalence of images lacking pedestrian information, a filtering process was necessary. Consequently, out of an initial pool of tens of thousands, a total of 5546 city pedestrian images were successfully acquired. The division between training and validation sets is at an 8:1 ratio.

**Figure 8:**
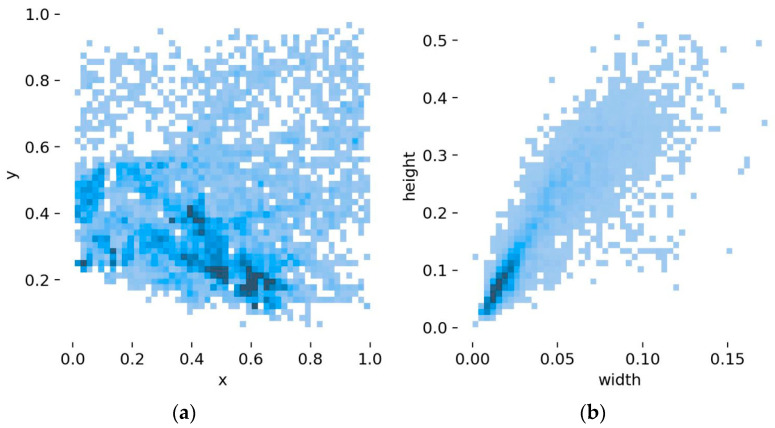
Data visualization. (**a**) The location of object boxes. (**b**) The size of object boxes.

Figure 8 provides a visual representation of the dataset, depicting the size and distribution of the pedestrians. Figure 8a displays the distribution of the centroid of the pedestrian detection frame after normalizing the image size. The figure reveals that pedestrians are predominantly situated in the lower–middle portion of the image. Figure 8b portrays details concerning the ratio of the object detection frame to the image size. Evidently, a significant portion of the target pedestrians appear to be distant within the images, with the majority being smaller targets. This insight from the dataset contributes to the refinement of the network structure for optimal performance.

### 3.2. Retinex Enhancement Algorithm

**Figure 9:**
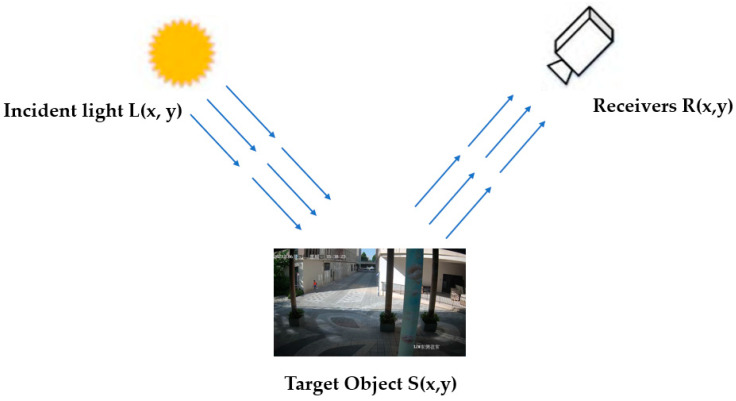
Principle of Retinex algorithm.

During the process of image acquisition, the inherent device components and environmental conditions can potentially result in image quality degradation. To tackle this issue, Retinex image enhancement technology is implemented as a solution. This technology emulates the human visual system by portraying the relative reflectance of objects in diverse lighting contexts. Leveraging the concept of color permanence intrinsic to the human visual system, the Retinex theory is strategically applied to address these considerations [32]. The principle of the Retinex is shown schematically in Figure 9.

The algorithm’s pivotal attribute lies in its adeptness at harmonizing color constancy, edge enhancement, and dynamic range compression [33], breaking the limitation that traditional linear or nonlinear can only be enhanced on one type of image feature. The Retinex algorithm is calculated using Formulas (7)–(9).

(7) $$R_{i}=logS_{i}\left(x,y\right)-log[F\left(x,y\right)*S_{i}\left(x,y\right)]$$

(8) $$F\left(x,y\right)=Ke^{-(x^{2}+y^{2}/c^{2})}$$

(9) $$\iint F\left(x,y\right)dxdy=1$$

where $R_{i}(x,y)$ denotes the object image enhancement output with subscripts; i∈R, G, and B, which represent the red, green, and blue three color bands, respectively; $R_{i}(x,y)$ denotes the distribution of the image $S\left(x,y\right)$ in the spectral band; * is the convolution operation; and $F\left(x,y\right)$ is the normalized surround function, which is the standard deviation of the Gaussian function. The surround function is a key component of Retinex-based algorithms, which aim to enhance the perceived quality of an image by adjusting the pixel values based on their local context.

**Figure 10:**
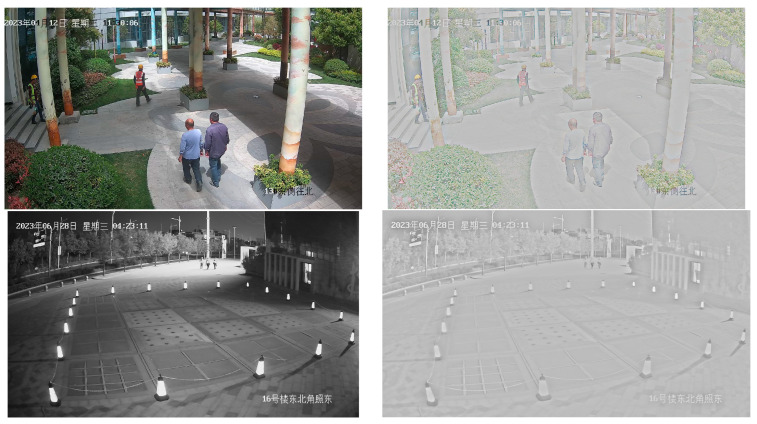
Source image and Retinex image.

Upon Retinex application, as depicted in Figure 10, the image showcases a heightened emphasis on texture features, while discarding extraneous attributes. This approach mitigates interference from irrelevant traits, allowing the model to concentrate on texture features for an enhanced performance. However, during nighttime, the output image is rendered in grayscale, which can impact the performance of the Retinex algorithm.

### 3.3. Image Enhancement

The model’s training was hindered by the limited data collected under constrained conditions, leading to potential underfitting or overfitting problems and significant impacts on the accuracy. To counteract this limitation, image enhancement techniques were employed to augment the dataset [34]. Primarily, denoising the images, which often exhibit noise due to the image capture process, serves to enhance their quality. Furthermore, to acknowledge the impact of real-world environmental conditions, image enhancement involves adjustments to the brightness and contrast. This strategy equips the model with the ability to adapt to diverse urban environments, bolstering its robustness. Images undergo a cropping operation, a technique that generally amplifies the model’s performance and facilitates reasonably accurate recognition even when images lack comprehensive data information.

### 3.4. Loss Function

The performance of the model is directly influenced by the loss function. In YOLOv5, the loss can be categorized into three components: Class_loss, Confidence_loss, and Location_loss. Specifically, confidence loss is computed solely when a specific bounding box lacks a target. Conversely, if a target is present, the ultimate loss encompasses the aggregate of all the aforementioned components. Notably, the current default choice for position loss in YOLOv5 is CIOU [35]. However, there are some problems with CIOU:

- Under certain circumstances, it will make the proportional consistency parameter of the aspect ratio of the predicted frame and the rear frame be 0, which makes the loss function unable to make a practical judgment.
- When the length and width of the anchor box increase and decrease at the same time, respectively, it will make the predicted box unable to fit the actual box effectively.
- The aspect ratio of the calculated box cannot effectively reflect the gap between the anchor box and the existing box.

Focal-EIOU [36] is used in this experiment instead of CIOU, and Focal-EIOU is calculated using Formulas (10)–(12).

(10) $$L_{EIOU}=L_{IOU}{+L}_{dis}+L_{asp}=1-IOU+\frac{\rho^{2}\left(b,b^{gt}\right)}{c^{2}}+\frac{\rho^{2}\left(w,w^{gt}\right)}{c_{w}^{2}}+\frac{\rho^{2}\left(h,h^{gt}\right)}{c_{h}^{2}}$$

(11) $$L_{f(x)}=\left\{\begin{array}{cc} \propto x^{2}\left[2\ln\left(\beta x\right)-1\right], & 0<x\leq 1;1/e\leq \beta \leq 1\\ -aln\left(\beta \right)x+C, & x>1;1/e\leq \beta \leq 1 \end{array},\right.$$

(12) $$L_{Focal-EIOU}=IOU^{\gamma }L_{EIOU}$$

where x denotes the difference between the actual values and predicted; β is used to control the curve’s arc; e is a natural constant; γ is used to control the degree of outlier suppression; and C is a constant. EOU improves CIOU by dividing the loss function into the distance, direction, and IOU loss. $C_{w}$ and $C_{h}$ are the length and height of the minimum box covering the actual box and anchor box, which solves the aspect ratio problem of CIOU.

Surveillance equipment is susceptible to various factors, including environmental conditions and weather, which can lead to fluctuations in the image quality. For instance, during the evening, the captured images might exhibit lower quality, with pedestrians’ silhouettes appearing unclear. Such scenarios can potentially lead to the model generating false alarms and leaks. CIOU enables the swift identification of evident samples, but falls short in recognizing challenging instances effectively. Hence, to enhance the model’s pedestrian recognition accuracy, distinct gradients are assigned to the FocalL1 loss, as depicted in Equation (8). A heightened gradient is established in regions with substantial error rates, directing the model’s attention towards challenging samples. The ultimate Focal EIOU loss is attained through the amalgamation of the EIOU loss and FocalL1 loss, as demonstrated in Equation (9). This alignment renders Focal EIOU as a well-suited fit for both the pedestrian scene within smart cities and the YOLO algorithm. Enhancing the YOLOv5s model through the integration of this loss function will elevate the model’s accuracy, while fortifying its robustness.

### 3.5. Evaluation Criteria

For the comprehensive evaluation of the model’s performance, Recall (R), precision (P), and mean average precision (mAP) are the commonly employed metrics. Given the experiment’s emphasis on the time taken for simultaneous detection using multiple cameras, accomplishing this experimental scenario necessitates defining simultaneous detection for the 50-camera setup. This involves summing the detection time for each frame of this model and calculating the average time consumption across 100 inferences to yield the final data results. The formulas for calculating the model evaluation indices are represented by Formulas (13)–(15).

(13) $$P=\frac{TP}{TP+FP}$$

(14) $$R=\frac{TP}{TP+FN}$$

(15) $$mAP=\frac{\sum_{j=1}^{N}\int_{0}^{1}PdR}{N}$$

where TP is the amount of correctly detected pedestrians; FP is the amount of incorrectly detected pedestrians; FN is the number of missed pedestrians; and N denotes the category.

## 4. Results and Discussion

### 4.1. Experimental Environment

The authors used PyTorch deep learning framework based on Python language; hardware environment: CPU is i7-13700k @5.40GHZ; GPU is Nvidia GeForce RTX 3070; OS is Window11; Python version is 3.8. compiled by PyCharm; and Tensorboard was used to visualize the experimental results. All the experimental results below were obtained using the platform built above. Table 1 provides training parameters for the YOLO models used.

### 4.2. Model a Priori Box Clustering

The size of the anchor box significantly influences the model’s performance and convergence speed. An anchor box is a predefined bounding box with a specific size and aspect ratio. These anchor boxes are used during object detection tasks. Anchor boxes were placed at various positions within an image and at different scales and aspect ratios to help the model detect objects of varying sizes and shapes. During training, the model learns to predict the offsets and class probabilities associated with these anchor boxes. These predictions enable the model to identify and locate objects in an image by matching the anchor boxes with the detected objects based on their characteristics.

In this study, the K-means algorithm was applied to our custom dataset, resulting in nine sets of anchor boxes of varying sizes. Small-sized anchor boxes were utilized on larger-scale feature mAP to detect small targets, while large-sized anchor boxes were employed on smaller-scale feature mAP to detect larger targets. The allocation of the nine sets of anchor boxes to the three different scales of detection feature layers in the YOLOv5-MS model is presented in Table 2.

### 4.3. Ablation Experiments

We employed a variety of strategies to improve the performance of the pedestrian detection system in the smart city. The ablation experiments have confirmed the efficacy of the aforementioned techniques (enhanced BackBone structure, Retinex image augmentation, K-means, SE, and Focal-EIOU). In the table, “√” denotes the utilization of the mentioned improvements, while “-” indicates that the technique was not applied. Simultaneously, the techniques mentioned above were employed in a non-sequential manner, as they pertain to distinct modules and operated independently of one another. Model training commenced only after the application of these techniques. We assessed the model’s performance through the mAP parameter and the model’s inference speed, considering the practical requirements of the smart city. The outcomes are detailed in Table 3.

### 4.4. Comparison Experiment

**Figure 11:**
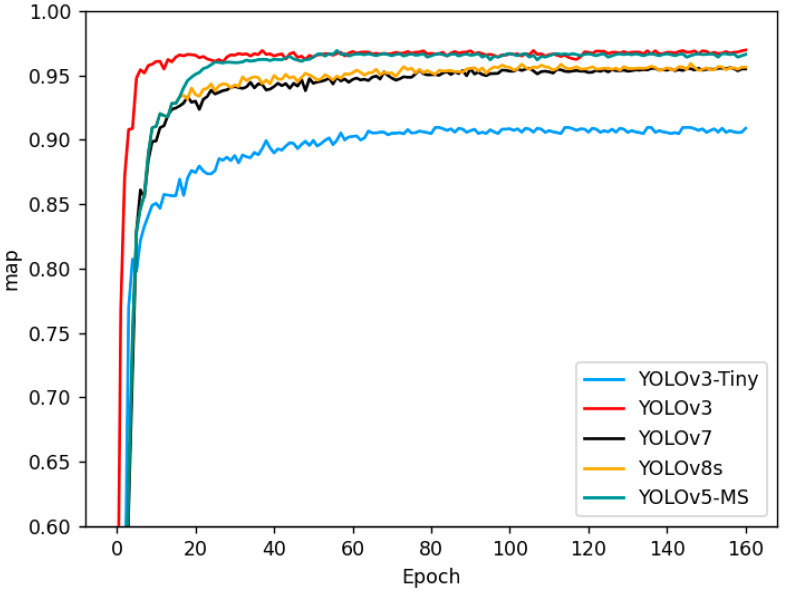
Comparison of training parameter trend.

To further ascertain the model’s performance, the YOLOv5-MS model was subjected to a comparison with YOLOv3-Tiny, YOLOv3, YOLO7, and YOLOv8s. The models were evaluated using consistent hyperparameters and training parameters. This study aims to identify a pedestrian target detection algorithm suitable for extensive camera usage in smart cities, striking a balance between accuracy and speed. The mAP trends of the five models over the initial 170 epochs are illustrated in Figure 11. All the models were simultaneously evaluated for their inference time on the platform designed as described above, while the ultimate outcomes are presented in Table 4. The results indicate that YOLOv5-MS exhibits a commendable mAP and inference speed performance.

### 4.5. Discussion

#### 4.5.1. Ablation Experiments Discussion

Table 3 presents notable findings. When exclusively utilizing the RepvggBlock module, the model’s mAP decreases in comparison to that of YOLOv5s, while its inference speed increases. This trend suggests that the module enhances the model’s inference speed at the expense of performance. However, upon integrating Focal-EIOU, the model’s mAP shows an improvement without significantly affecting the inference speed. The efficacy of the K-means clustering algorithm is also evident in this experiment, implying that it successfully enhances the compatibility between Focal-EIOU and both the smart city pedestrian scene and the YOLO algorithm, thereby improving the model’s overall performance. The enhanced accuracy attributed to the Retinex algorithm can be attributed to its capability to aid the model in focusing on texture features, which is a critical aspect in the target detection of pedestrians within urban settings. The incorporation of the SE attention module within the network has led to a 0.4% improvement in the mAP of the model, signifying that this module successfully extracted pertinent information that contributed to enhancing the model’s performance. The inference speed of YOLOv5-MS outpaces that of YOLOv5s by 21.3%, showcasing the efficiency gained through these improvements. Each of these enhancements collectively attests to the scientific validity and effectiveness of the upgraded model.

**Figure 12:**
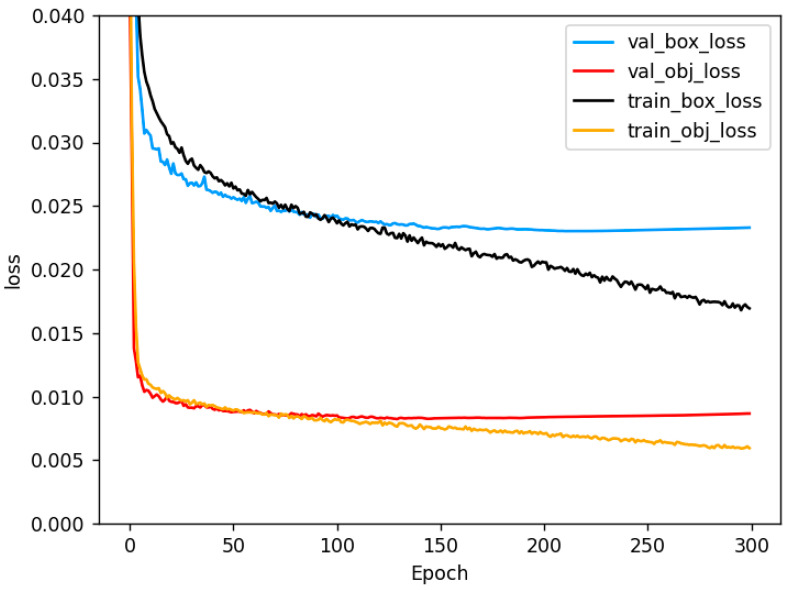
Loss curve.

Figure 12 displays the training progress of the YOLOv5-MS model over 300 epochs, illustrating both a training loss curve and a validation loss curve. The horizontal axis represents the epoch, while the vertical axis represents the corresponding loss value. It is noticeable that the loss function of the training set steadily decreases over the epochs. However, the confidence loss of the validation set begins to exhibit a gradual increase after approximately 160 epochs. Moreover, the loss related to border regression starts to stabilize, suggesting a potential tendency towards overfitting in the model. Consequently, training could be halted around the 160th epoch.

**Figure 13:**
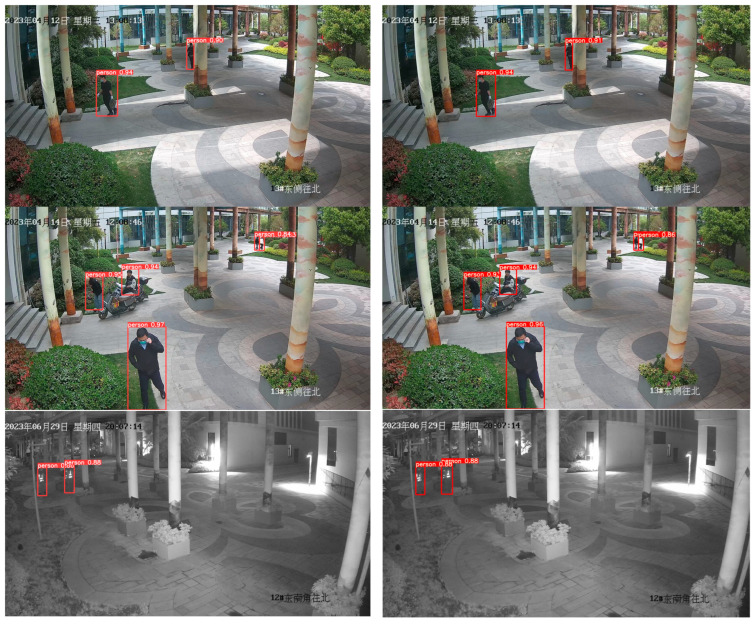

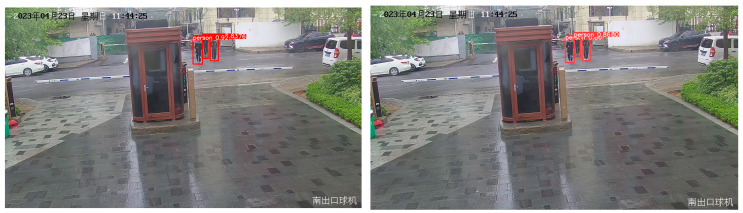
First column: YOLOv5 inference results. Second column: YOLOv5-MS inference results.

Figure 13 displays a series of comparative plots for different scenarios, with the first column depicting the inference results for the YOLOv5s model and the second column presenting the inference results for the YOLOv5-MS model. The first row illustrates that in unobstructed pedestrian scenarios, both the YOLOv5-MS and YOLOv5s algorithms perform the proficient detection of pedestrian targets, with comparable confidence levels, ensuring accurate detection. Moreover, YOLOv5-MS showcases a slightly superior confidence level in detecting occluded targets. In the second row, YOLOv5-MS demonstrates the better detection of small targets compared to that of YOLOv5s, although YOLOv5s excels in detecting larger targets. The third row showcases a uniform performance between the two models in detecting pedestrians during nighttime. Finally, in the fourth row, YOLOv5s exhibits higher confidence in detecting conspicuous pedestrians in rainy conditions, while YOLOv5-MS demonstrates a superior capacity to generalize the detection of multiple small targets. Based on the observations above, it is evident that the predominant nature of the pedestrian targets in this scenario involves small target detection. Therefore, a model specialized in robustly detecting small targets should be utilized, rendering the YOLOv5-MS model more suitable for this experimental setting. In summary, YOLOv5-MS demonstrates a superior performance over that of YOLOv5s in detecting occluded or small targets, but falls behind YOLOv5s in detecting larger targets.

#### 4.5.2. Comparison Experiments Discussion

The comparative experiments presented in Table 4 reveal an interesting trade-off between the model’s mAP and its inference speed. This relationship is characterized by an inverse correlation; when the weight is increased, the mAP value tends to rise while the inference speed decreases, and conversely, when the weight is decreased, the inference speed increases, but the mAP value tends to decline. In this experiment, YOLOv3 exhibits a 0.4% higher mAP value compared to that of YOLOv5-MS. However, YOLOv3 has a significantly larger number of parameters than YOLOv5-MS does, resulting in YOLOv3’s inference time being more than twice as long as that of YOLOv5-MS. Specifically, using the YOLOv3 model, it requires 0.975 s to simultaneously process each frame from 50 cameras. Conversely, when employing YOLOv5-MS, this processing time is reduced to 0.416 s to achieve the same results. Consequently, if we utilize YOLOv5-MS to detect 100 cameras with adequate hardware capabilities, it would take the same duration as YOLOv3’s 50-camera detection, while maintaining a comparable accuracy. This translates to nearly halving the overall cost.

While YOLOv3-Tiny boasts an almost halved inference speed compared to that of YOLOv5-MS, its mAP value falls short of our requirements. It is important to note that we cannot indefinitely reduce costs without ensuring accuracy. Furthermore, when compared to YOLOv7 and YOLOv8, they do not match YOLOv5-MS in terms of both speed and mAP. YOLOv5-MS, on the other hand, achieves a remarkable feat by performing simultaneous detection using 50 cameras in just 0.416 s, all while maintaining an impeccable accuracy. This exceptional model performance positions it as the ideal choice for target detection applications in smart cities.

## 5. Conclusions

This paper introduces an advanced pedestrian target detection model, referred to as YOLOv5-MS. It is built upon the YOLOv5 architecture and specifically designed for robust pedestrian target detection within a smart city context. Initially, YOLOv5 serves as a foundational framework, ensuring the efficacy of target detection performance. Subsequently, optimization is applied to the acquisition of multiple video streams within the model. To further enhance efficiency, RepvggBlock is introduced into the BackBone segment, replacing the original convolutional layer, and thereby, expediting the model’s inference process. To enrich the model’s capabilities, an SE attention module is integrated into the network, enabling the extraction of more pertinent information from images and, consequently, augmenting the overall model performance. This integrated approach ensures the advancement of pedestrian target detection within the context of smart cities. The model’s performance gains are also attributed to the integration of the K-means algorithm and the Retinex image enhancement technique. Additionally, the adoption of the Focal EIOU loss, replacing the previous CIOU loss, contributes to the model’s advancement. These enhancements are methodically validated through ablation experiments. The experimental outcomes demonstrate a notable enhancement of the model’s mAP by 2.0%, accompanied by a substantial 21.3% improvement in inference speed compared to that of the original model. Furthermore, the model’s superiority is highlighted by its outperformance when compared to the performances of the other state-of-the-art models. These discoveries have led to improved applications for pedestrian target detection using large-scale cameras in smart city environments.

Nevertheless, there is untapped potential for further improvement in this model. In the forthcoming research endeavors, we intend to explore the opportunities for optimizing the model’s structure, while upholding the detection accuracy. This optimization aims to enable simultaneous detection across a larger number of cameras, rendering the model more suitable for pedestrian detection within complex urban environments. By fine-tuning the model’s architecture, we aspire to bolster its applicability and performance in the dynamic and intricate landscapes of smart cities. At the same time, we will focus on multi-target model detection, a research direction that will empower us to address the diverse challenges within smart cities with a combination of high accuracy and cost-effectiveness.

## Figures and Tables

**Table 1:** Training parameters of models.

Parameters Items	Value
Epoch	300
Batch size	16
Worker	8
Momentum	0.937
Initial learning rate	0.01
Input size	640 × 640
Weight decay	0.0005

**Table 2:** Priori box data.

Scale	Priori Framework		
20 × 20	[42.22, 85.29]	[45.36, 112.81]	[63.04, 125.6]
40 × 40	[17.35, 47.73]	[24.29, 63.40]	[30.37, 87.94]
80 × 80	[6.66, 15.85]	[9.65, 26.26]	[13.33, 35.21]

**Table 3:** Ablation experiment results.

RepvggBlock	Focal-EIOU	K-Means	Retinex	SE	mAP	Speed
-	-	-	-	-	94.5%	0.529 s
√	-	-	-	-	93.9%	0.401 s
√	√	-	-	-	94.6%	0.408 s
√	√	√	-	-	95.4%	0.406 s
√	√	√	√	-	96.1%	0.411 s
√	√	√	√	√	96.5%	0.416 s

**Table 4:** The comparison among different proposed models.

Model	P	R	mAP	Speed	Parameter
YOLOv3-Tiny	92.1%	84%	90.8%	0.264 s	16.9 MB
YOLOv3	95.5%	94.1%	96.9%	0.975 s	117 MB
YOLOv7	95%	91.1%	95.5%	0.631 s	72 MB
YOLOv8s	94.6%	91.9%	95.6%	0.581 s	21.5 MB
YOLOv5-MS	95.9%	93.1%	96.5%	0.416 s	10.7 MB

## Data Availability

This data comes from the Zhengtou City in Zhengzhou Henan.
